# Metabolic profiling as a powerful tool for the analysis of cellular alterations caused by 20 mycotoxins in HepG2 cells

**DOI:** 10.1007/s00204-022-03348-5

**Published:** 2022-08-06

**Authors:** Andrea Gerdemann, Matthias Behrens, Melanie Esselen, Hans-Ulrich Humpf

**Affiliations:** Institute of Food Chemistry, University of Münster, Corrensstraße 45, 48149 Münster, Germany

**Keywords:** Metabolomics, Mycotoxins, Mode of action, HepG2, Metabolic profiling, HILIC

## Abstract

Mycotoxins are secondary fungal metabolites which exhibit toxic effects in low concentrations. Several mycotoxins are described as carcinogenic or immunosuppressive, but their underlying modes of action especially on molecular level have not yet been entirely elucidated. Metabolic profiling as part of the omics methods is a powerful tool to study the toxicity and the mode of action of xenobiotics. The use of hydrophilic interaction chromatography in combination with targeted mass spectrometric detection enables the selective and sensitive analysis of more than 100 polar and ionic metabolites and allows the evaluation of metabolic alterations caused by xenobiotics such as mycotoxins. For metabolic profiling, the hepato-cellular carcinoma cell line HepG2 was treated with sub-cytotoxic concentrations of 20 mycotoxins. Moniliformin and citrinin significantly affected target elements of the citric acid cycle, but also influenced glycolytic pathways and energy metabolism. Penitrem A, zearalenone, and T2 toxin mainly interfered with the urea cycle and the amino acid homeostasis. The formation of reactive oxygen species seemed to be influenced by T2 toxin and gliotoxin. Glycolysis was altered by ochratoxin A and DNA synthesis was affected by several mycotoxins. The observed effects were not limited to these metabolic reactions as the metabolic pathways are closely interrelated. In general, metabolic profiling proved to be a highly sensitive tool for hazard identification in comparison to single-target cytotoxicity assays as metabolic alterations were already observed at sub-toxic concentrations. Metabolic profiling could therefore be a powerful tool for the overall evaluation of the toxic properties of xenobiotics.

**Supplementary Information:**

The online version contains supplementary material available at 10.1007/s00204-022-03348-5.

## Introduction

Metabolomics methods deal with the analysis of the smallest compounds (< 1500 Da) in a biological system and complement the omics methods on metabolite level (Halama 2014). The analysis of major metabolic pathways allows to get an overview as well as deeper insights into cellular mechanisms at the metabolite level that are closely linked to the phenotype (Bujak et al. 2015). In recent years, metabolic profiling gained increasing interest in toxicological research as a new approach for hazard characterization in food and feed safety.

Mycotoxins are secondary metabolites of fungi from various species that often exhibit toxic effects at low concentrations. Some fungal species like *Aspergillus* and *Penicillium* can infest food and feed post-harvest, whereas *Fusarium* grows on the fields and infests the crops itself. Under certain conditions, this infestation leads to mycotoxin contamination, which makes toxicological evaluations mandatory (Alshannaq and Yu 2017). More than 300 mycotoxins with broad structural diversity have been identified causing various toxic effects of these substances. Effects like immunosuppression, nephrotoxicity, mutagenicity, and carcinogenicity are commonly described for mycotoxins. Several toxicity endpoints have been studied in detail in animal studies, but data on distinct cellular response and the mode of action are still scarce (Wen et al. 2016).

For metabolic profiling, nuclear magnetic resonance spectroscopy (NMR), gas chromatography, as well as high-performance liquid chromatography coupled with mass spectrometric techniques (GC–MS, HPLC–MS) are commonly used (Bujak et al. 2015). In contrast to NMR spectroscopy, MS detection allows for multiplexing, simultaneously quantifying a large number of compounds with different concentrations. In addition, the LC–MS coupling is suitable for a wide range of analytes and polarities without any derivatization, which is a key advantage over GC–MS methods. Since many relevant cellular metabolites are highly polar or even ionic, the development of a chromatographic separation method is challenging. The most commonly used reversed stationary phases are not suitable for this kind of analytes due to their low retention. Hydrophilic interaction chromatography (HILIC) is the complementary technique which enables the separation of small polar and charged analytes. Compared with silica-based HILIC columns, zwitterionic stationary phases are suitable for wider ranges of analytes and achieve more reproducible chromatographic results (Sonnenberg et al. 2019).

To study the effects of mycotoxins on the human meta-bolome, an HILIC-MS/MS method was developed, which includes more than 100 metabolites of the main cellular metabolic pathways. Twenty different mycotoxins from different fungal species like *Penicillium*, *Fusarium*, *Aspergillus*, *Alternaria*, *Stachybotrys*, and *Claviceps* were selected for metabolic profiling analysis. From each class of secondary metabolites, the most toxic compound (based on literature data) was selected and human liver cancer HepG2 cells representing the target organ of xenobiotic metabolization were treated with sub-toxic concentrations.

## Materials and methods

### Chemicals and reagents

The solvents were purchased in LC–MS grade from Carl Roth (Karlsruhe, Germany), Fisher Scientific (Schwer-te, Germany), or Sigma-Aldrich (Steinheim, Germany). Ammonium acetate was used from VWR (Darmstadt, Germany) and ammonia (25 vol%) was obtained from Grüssing (Filsum, Germany). Purified water was generated using a PureLab Flex2 system (Veolia Water Technologies, Celle, Germany). As internal standard for metabolic profiling d-(-)-α-phenylglycine was used, which was purchased from Sigma-Aldrich (Steinheim, Germany) in 99% purity. The concentrations, chemical structures and purities of the selected mycotoxins are defined in Online-Resource 1 (Figure S1, Table S1).

### Cell culture

The human hepatocellular carcinoma cell line HepG2 (ATCC, Manassas, USA) was cultured in Dulbecco´s Modified Eagle Medium (DMEM, high glucose, with glutamine, Gibco, Prat de Lloregat, Barcelona, Spain) supplemented with 10 mM N-2-hydroxyethylpiperazine-*N*´-2-ethanesulfonic acid (HEPES buffer, Carl Roth, Karlsruhe, Germany), 10% (v/v) fetal calf serum (FCS, PAN Biotech, Aidenbach, Germany) as well as 100 U/mL penicillin and 100 µg/mL streptomycin (PAN Biotech, Aidenbach, Germany) as antibiotics. The cells were cultured in humidified atmosphere with 5% CO_2_ and were subcultured after trypsinization every 7 days. The cell culture medium was exchanged twice a week.

### Sample preparation for metabolic profiling

HepG2 cells were seeded in cell culture dishes with 6 cm diameter (Sarstedt, Nümbrecht, Germany) at 1.2 × 10^6^ cells/plate. The DMEM containing FCS was replaced by serum-free medium after 24 h of growing. After 24 h, mycotoxins were added in sub-toxic concentrations, which had been determined using the resazurin reduction assay. The cytotoxicity assay was performed according to a previous publication (O'Brien et al. 2000) and the results are shown in the Online-Resource 1 (Figure S2). After 24 h of treatment, the cell culture medium was removed and the cells were washed twice using sterile phosphate buffered saline (PBS, PAN Biotech, Aidenbach, Germany). PBS was removed and 400 µL pre-cooled (8 °C) acetonitirile (ACN)/water (4 + 1, *v*/*v*) including 50 µM phenylglycine as internal standard for sample preparation was added immediately for metabolic quenching. The solvent was evenly spread on the surface and the cell culture dishes were stored at 8 °C until further sample preparation. The cells were detached using a sterile cell scraper (Fisher Scientific, Schwerte, Germany) and the cell culture dish was washed twice with pre-cooled ACN/water (4 + 1, *v*/*v*) without internal standard. The cell suspensions were combined in sterile 2 mL Safe-Lock tubes (Eppendorf, Wesseling, Germany) and treated 15 min in an ice-cooled ultrasonic bath for cell extraction. Afterwards, the suspension was centrifuged (15 min, 4 °C, 14,840 × g). The supernatant (cell extract) was used for metabolic profiling and the precipitate (cell pellet) preserved for the determination of the deoxyribonucleic acid (DNA) concentration to estimate the cell count using GelGreen (Merck, Darmstadt, Germany) as DNA intercalating dye. The results and detailed sample preparation for DNA determination are shown in the Online-Resource 1 (Figure S3). The cell extract was dried under vacuum, reconstituted in 80 µL ACN/water (1 + 1, *v*/*v*), and sonicated again. In case of insoluble residues, the tubes were centrifuged again (15 min, 20 °C, 14,840 × g) and the supernatant was used for LC–MS measurements. For each test compound, three indepen-dent experiments were performed in duplicate (*n* = 3 × 2).

### Chromatographic and mass spectrometric conditions

For chromatographic separation, a peek-lined InfinityLab Poroshell 120 HILIC-Z column (2.1 mm × 100 mm, 2.1 µm, Agilent Technologies, Waldbronn, Germany) was used with a 15 min gradient elution of ACN and water. Solvent A was a mixture of ACN/water (19 + 1, *v*/*v*) and as solvent B ACN/water (1 + 1, *v*/*v*) was used. Both solvents were supplemented with 10 mM ammonium acetate and were adjusted to pH 9 using ammonia (25 Vol%). Detailed HPLC parameters are presented in Online-Resource 1. For method development, a quadrupole time-of-flight hybrid mass spectrometer equipped with an Apollo II source (Impact II, Bruker Daltonics, Bremen, Germany) was used and for targeted analysis a triple quadrupole mass spectrometer (EVOQ Elite, Bruker Daltonics, Bremen, Germany) with an HESI source (heated electrospray ionization) was selected. The mass spectrometers were both equipped with an Elute HAT Pump HPG 1300 (Bruker Daltonics, Bremen), an Elute Column Oven (Bruker Daltonics, Bremen, Germany), and a PAL HTC-xt auto sampler (CTC analytics, Zwingen, Switzerland). The specific transitions for each analyte were picked from fragmentation spectra of previous high-resolution mass spectrometry (HRMS) measurements or public spectral libraries like the Human Metabolome Database (HMDB, Wishart et al. 2018) and the Mass Bank of North America (MoNA, https://massbank.us/). The optimization of collision energies (CE) for the targeted HPLC–MS/MS measurement was performed software-aided using Skyline (Version 21.1, University of Washington, (Adams et al. 2020)). The evaluated CE values ranged from 5 to 50 eV with a step size of 5 eV. The final number of MRM (multiple reaction monitoring) transitions was reduced, to get the most scan time for each data point and maximize the sensitivity. The protocol for HPLC method optimization and the source parameters are available in the Online-Resource 1 and the final transitions are listed in the Online-Resource 2 (Table S3).

### Data processing and statistical analysis

For method development, the HRMS data were processed using Compass Data Analysis 4.4 (Bruker Daltonics, Bremen, Germany) and Skyline 21.1. For metabolite identification via Metaboscape 5.0 (Bruker Daltonics, Bremen, Germany), the comparison of retention time, exact mass, isotopic pattern, and fragmentation spectra were used. The fragmentation spectra were compared with reference substances or public spectral libraries. Available reference compounds are listed in Online-Resource 2 (Table S3).

The targeted analyses were processed using MS Workstation (Version 8.2.1, Bruker Daltonics, Bremen) and TASQ 2.2.14 (Bruker Daltonics, Bremen, Germany). The retention times were compared with quality control measurements of a reference cell extract and a combined standard solution of a variety of analytical compounds (Online-Resource 1, Table S2) included in each sample set. The reference cell extract was prepared by mixing extracts from different cell lines [IHKE (immortalized human kidney epithelial cells (Rottkord et al. 2017)), HT-29 (colon carcinoma cells (Müller et al. 2021)) and HepG2] to cover analytes with no available reference compounds. The integrated peak areas were further processed using Microsoft Excel 2019 (Microsoft Corporation, Redmond, USA). The metabolite peak areas were normalized to the internal standard peak area to compensate the loss of analytes during sample preparation. Afterwards each value was divided by the mean value of the solvent control samples in the same sample set and the fold change was calculated and normalized to zero. Due to the lack of reference compounds for all analytes, the results are semi-quantitative and the cellular changes were calculated relative to the solvent control. The mean and the relative standard deviation of all replicates were calculated, and for statistical analysis, an unpaired, heteroscedastic Student’s T test was performed. Bar graphs, the principle component analysis (PCA), and the heatmap were prepared using Ori-ginPro 2022 (OriginLab Corporation, Northampton, MA, USA).

As the isomeric triose phosphates glyceraldehyde 3-phosphate and dihydroxyacetone phosphate as well as the pentose phosphates ribose 5-phophate, ribulose 5-phosphate, and xylulose 5-phosphate were not separated chromatographically, their peak areas were summarized as triose phosphate (triose-P) and pentose phosphate (pentose-P) in the following.

## Results and discussion

### Method development

For metabolic profiling, a targeted HILIC–MS/MS method was developed covering more than 100 metabolites of the main cellular metabolic pathways. The detailed development parameters are described in Online-Resource 1 (Figure S4 and S5). In short, the replacement of a stainless steel capillary by PEEK (polyetheretherketone) material was crucial to reduce tailing of organic acids and phosphates. Both classes of metabolites suffered from short-term interaction with metal ions as long as stainless steel capillaries are installed (Tuytten et al. 2006; Shi et al. 2002). Therefore, the choice of the LC–MS system and the in-built stainless steel components also significantly affected the chromatographic results. The combination of an Elute pump and an EVOQ mass spectrometer generated symmetric peak shapes and enabled reproducible peak integration, which might be due to less stainless steel components in the system. The use of a peek-lined zwitterionic HILIC column compared to a silica-based HILIC column further improved the peak shape of a variety of analytes as zwitterionic phases are well suited for very polar and ionic analytes. The variation of the pH value had only minor effects on the peak shape but alkaline conditions were selected because of less corrosive effects and better ionization conditions for anionic analytes at higher pH values (Tuytten et al. 2006).

### Metabolic effects caused by mycotoxins

**Fig. 1:**
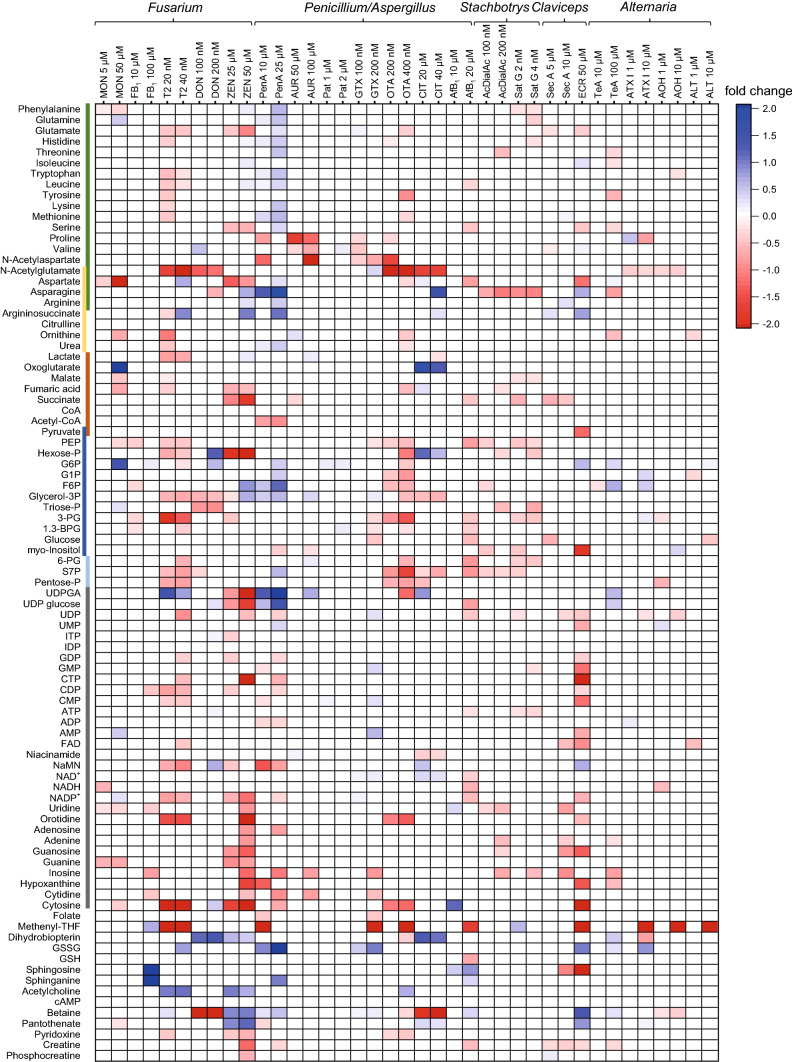
Heatmap of significant metabolic alterations (Student’s *T* test, *p* ≤ 0.01, *n* = 3 × 2) caused by different mycotoxins in HepG2 cells after 24 h and illustrated as fold change to solvent control. Red boxes indicate a depletion of analytes relative to the solvent control and blue boxes indicate an enrichment. Insignificant alterations are not colored. The color code at the left side assigns the analytes to different metabolic pathways and structural classes: amino acids, urea cycle, citric acid cycle, glycolysis,pentose phosphate pathway,nucleoside derivatives (color figure online)

**Fig. 2:**
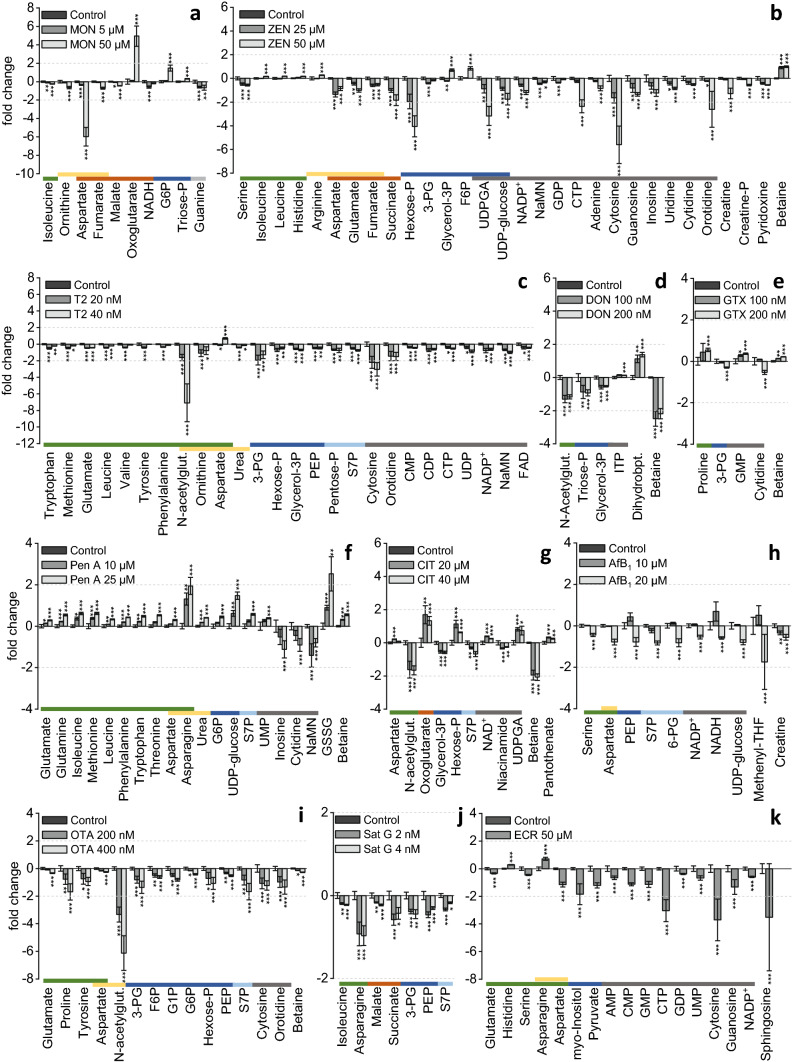
Highly significant metabolic alterations caused by selected mycotoxins illustrated as bar graphs. The values are calculated as fold change in comparison to solvent control including the respective significance levels ***p ≤ 0.001, **p ≤ 0.01, *p ≤ 0.05 according to Student’s *T* test (*n* = 3 × 2); **a** moniliformin (MON), **b** zearalenone (ZEN), **c** T2 toxin **d** deoxynivalenol (DON), **e** gliotoxin (GTX), **f** penitrem A (Pen A), **g** citrinin (CIT), **h** aflatoxin B_1_ (AfB_1_), **i** ochratoxin A (OTA), **j** satratoxin G (Sat G), and **k** ergocristine (ECR). The color code assigns the analytes to different metabolic pathways and structural classes. From the left to the right:  amino acids,urea cycle,citric acid cycle,glycolysis,pentose-phosphate pathway,nucleoside derivatives

**Fig. 3:**
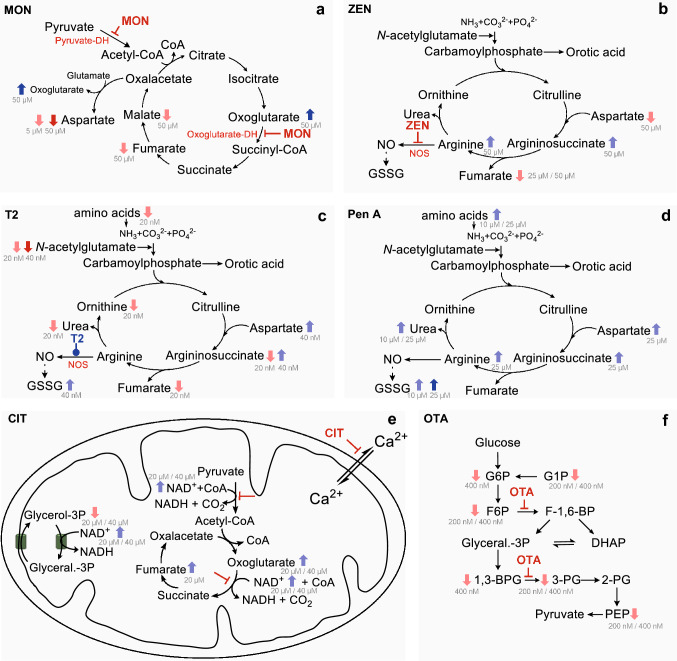
Biochemical pathways significantly (*p* ≤ 0.01) affected by selected mycotoxins; **a** moniliformin (MON), **b** zearalenone (ZEN), **c** T2 toxin, **d** penitrem A (Pen A), **e** citrinin (CIT), **f** ochratoxin A (OTA). Light blue arrows () illustrate an enrichment less than fold change of 2 and dark blue arrows ( ) illustrate an enrichment higher than twofold in the added test concentration. According to those, the light () and dark red ( ) arrows illustrate a depletion lower or higher than twofold.  illustrates a suggested inhibition of a specific enzyme and illustrates a suggested induction of a specific enzyme. *DH* dehydrogenase, *NOS* nitric oxide synthase

HepG2 cells were treated with twenty mycotoxins from different fungal species for 24 h. The sub-toxic concentrations were selected based on the results of the resazurin reduction assay (Online-Resource 1, Figure S2). The samples were analyzed via the above-mentioned HILIC-MS/MS method (for details see Materials and Methods and Online-Resource 1 and 2) and the results are summarized in a heatmap (Fig. 1) which gives an overview over all observed significant (*p* ≤ 0.01) metabolic effects caused by the investigated mycotoxins. Some mycotoxins such as Pen A, ZEN or T2 induced strong metabolic effects, whereas others like PAT or *Alternaria* toxins affected the metabolome just slightly. In general, many mycotoxins had significant effects on the metabolome which could be related to their cytotoxic potential. The results of specific mycotoxins are presented and evaluated as follows. The fold changes were calculated relative to the solvent control, because references were not available for all analyzed analytes. All of the highly significant metabolic effects (p ≤ 0.001) caused by at least one concentration of selected mycotoxins are discussed in the text and visualized as bar graphs in Fig. 2. Cellular alterations with lower significance (p ≤ 0.01, p ≤ 0.05) were explained in the text if their results fit to the context of effects with higher significance or literature data. For better comparability of the values, also small fold changes are mentioned. All of the assigned numeric values in the text reached a significance level of at least p ≤ 0.05 in at least one concentration if not stated otherwise. The bar graphs of additional mycotoxins are shown in Online-Resource 1 (Figure S6) and the numeric fold changes in combination with standard deviation and statistical significance of all metabolites are shown in Online-Resource 2 (Table S4). The alterations (p ≤ 0.01) of the specific metabolic pathways are illustrated in Fig. 3. Additionally, a PCA plot including all affected metabolites can be found in Online-Resource 1 (Figure S7).

### Metabolic effects caused by *Fusarium* mycotoxins

Secondary metabolites produced by *Fusarium* species include fumonisins, trichothecenes, zearalenone (ZEN), and a variety of additional mycotoxins such as moniliformin (MON) or enniatins which belong to the so-called emerging mycotoxins (Marin et al. 2013).

### Moniliformin

HepG2 cells were treated with MON at concentrations of 5 µM and 50 µM. This mycotoxin-induced significant alteration of the citric acid cycle as a strong accumulation of oxo-glutarate (4.9-fold) was observed after testing of 50 µM MON (Figs. 2a and 3a). In addition, organic acids such as succinate (-0.3-fold), fumarate (-0.7-fold) and malate (-0.4-fold) which are also metabolites of the citric acid cycle, were decreased significantly. The treatment with 5 µM MON also induced a significant decrease of nicotinamide adenine dinucleotide (NADH, -0.6-fold). These observations suggest that MON inhibits the oxoglutarate dehydrogenase, an enzyme converting oxoglutarate to succinyl-coenzyme A (succinyl-CoA). Previous publications on the mode of action of MON in cell free assays support these findings. MON has been described to inhibit pyruvate and oxoglutarate oxidation and to interact with thiamine pyrophosphate-dependent enzymes such as pyruvate dehydrogenase and oxoglutarate dehydrogenase (Thiel 1978; Pirrung et al. 1996). The increasing levels of glycolysis intermediates like glucose 6-phosphate (G6P, 1.5-fold) or triose-P (0.3-fold) after 50 µM treatment support this assumption due to the limited incorporation of pyruvate in the citric acid cycle. However, the level of pyruvate itself was not significantly affected. Further observations include a decrease of aspartate (sixfold, 50 µM), a metabolite connecting the citric acid cycle to the urea cycle. The accumulation of oxoglutarate could cause a product inhibition of the transamination of glutamate. Other metabolites of the urea cycle like ornithine (-0.6-fold) and argininosuccinate (-0.5-fold) were also significantly decreased by 50 µM MON, thus a decreased availability of aspartate and the linkage of citric acid cycle and urea cycle is supposed. The significant decrease of guanine in both concentrations (-0.6-fold/-0.7-fold) was not further evaluated.

### Fumonisin B_1_

The main observed effect of fumonisin B_1_ (FB_1_,10 µM and 100 µM) was the influence on the sphingosine metabolism. The sphinganine/sphingosine ratio increased significantly when cells were treated with 100 µM FB_1_. Sphinganine increased 105.3-fold and sphingosine increased 4.6-fold in comparison to solvent control (*p* ≤ 0.01). The same tendency was also observed at 10 µM FB_1_ but to a much lower extent. It has been previously described that FB_1_ effectively inhibits the ceramide synthase because of its structural similarities to the sphingosine backbone (Stockmann-Juvala and Savolainen 2008). As the ceramide synthase catalyzes the synthesis of ceramides from sphingosine (Levy and Futerman 2010) its inhibition leads to an accumulation of the substrates sphinganine and sphingosine. In addition to the FB_1_-related effects already described, an enrichment of cytidine monophosphate (CMP, 0.6-fold) and a decrease of cytidine diphosphate (CDP, -0.4-fold) which are also involved in lipid metabolism, was observed. Additionally, slight decreases in DNA and RNA (ribonucleic acid) building blocks like adenine (-0.3-fold) and adenosine (-0.6-fold), guanine (-0.6-fold) and guanosine (-0.3-fold), inosine (-0.7-fold), uridine (-0.4-fold), cytosine (-0.2-fold), and cytidine (-0.5-fold) were found after 100 µM testing, representing an impact on the DNA and RNA synthesis. For the first time, the results showed that 10 µM FB_1_ additionally influences the glucose metabolism by a decrease of fructose 6-phosphate (F6P, -0.3-fold), 1,3-bisphosphoglycerate (1,3-BPG, -0.2-fold), and phosphoenolpyruvate (PEP, -0.3-fold, Figure S6), which are intermediates of the glycolysis. These effects appear to be concentration-dependent as 100 µM FB_1_ was not effective.

### Zearalenone

HepG2 cells were treated with 25 µM and 50 µM ZEN for 24 h. In general, ZEN concentration dependently modulated different metabolic pathways (Fig. 2b). The concentration of DNA building blocks such as adenine (-0.2-fold/-0.8-fold), cytosine (-1.6-fold/-5.6-fold), guanosine (-0.8-fold/-1.3-fold), inosine (-0.6-fold/-1.2-fold), uridine (-0.4-fold/-0.8-fold), cytidine (-0.4-fold/-0.5-fold) and orotidine (-0.2-fold/-2.6-fold), as well as cellular DNA content (Figure S3) were significantly decreased by 25 µM and 50 µM ZEN, which may indicate an inhibition of the PPP (pentose phosphate pathway) or DNA synthesis. Additionally, the levels of aspartate (-1.4-fold/-0.9-fold), glutamate (-0.5-fold/-1.0-fold), succinate (-1.0-fold/-1.8-fold), and fumarate (-0.6-fold/-0.5-fold) involved in the citric acid cycle were reduced by ZEN at both concentrations. Oxoglutarate (0.4-fold, 25 µM) and acetyl-coenzyme A (acetyl-CoA, 0.6-fold/0.9-fold) were in contrast slightly enriched suggesting also an influence on cell energy metabolism. Glycolysis metabolites such as an unspecified hexose phosphate (hexose-P, -2.0-fold/-4.0-fold), 3-phosphoglycerate (3-PG, -0.4-fold/-0.2-fold), glycerol 3-phosphate (glycerol-3P, -0.2-fold/0.7-fold) and F6P (0.0-fold/0.8-fold) were also influenced. The glycolysis might be involved in the regulation of the energy production as the citric acid cycle seemed to be downregulated at this concentration. Uridine diphosphate glucose (UDP-glucose, -0.9-fold/-1.8-fold) and uridine diphosphate glucuronic acid (UDPGA, -0.9-fold/-3.2-fold), which are also involved in glucose metabolism, especially in gluconeogenesis, were also decreased. Furthermore, some analytes of the urea cycle like argininosuccinate (1.0-fold) and arginine (0.3-fold) were enhanced after 50 µM testing of ZEN (Fig. 3b). The metabolization of arginine via nitric oxide (NO) synthase also requires nicotinamide adenine dinucleotide phosphate (NADP^+^) as a cofactor and generates citrulline as a side product, which can be recycled as a substrate of argininosuccinate synthase. This cycle, which involves the generation of NO and the recycling of citrulline, is also known as citrulline-NO cycle. It has recently been published that ZEN inhibits the endothelial NO synthase in bovine aortic endothelial cells (Lee et al. 2020). An inhibition of this enzyme could have initiated the enrichment of arginine and argininosuccinate as well as the depletion of NADP^+^ (-0.6-fold/-1.2-fold).

The effects on the content of CMP (1.2-fold), CDP (-0.3-fold) and cytidine triphosphate (CTP, -2.4-fold) as well as the increase of sphinganine (2.3-fold) induced by 50 µM ZEN may be a result of effects on lipid metabolism. This effect is similar to the effects induced by FB_1_, which might indicate a slight inhibition of the ceramide synthase.

Further significant effects include an increase of betaine (0.9-fold/1.0-fold), isoleucine (0.2-fold, 50 µM) and histidine (0.2-fold, 50 µM) and a decrease of pyridoxine (-0.4-fold/-0.6-fold), creatine phosphate (-0.6-fold, 50 µM), creatine (-1.3-fold, 50 µM), guanosine diphosphate (GDP, -0.4-fold, 25 µM), nicotinic acid mononucleotide (NaMN, -0.5-fold, 25 µM) and serine (-0.5-fold/-0.6-fold).

### T2 toxin

The treatment of cells with 20 nM T2 toxin (Fig. 2c) decreased many amino acids such as glutamate (-0.5-fold), leucine (-0.4-fold), lysine (-0.3-fold), methionine (-0.5-fold), ornithine (-1.1-fold), phenylalanine (-0.4-fold), proline (-0.3-fold), tryptophan (-0.5-fold), tyrosine (-0.4-fold) and valine (-0.4-fold). T2 toxin´s impact on amino acid homeostasis could be connected to an inhibition of the protein biosynthesis which has been reported previously (Rosenstein and Lafarge-Frayssinet 1983; Pace et al. 1988). The treatment of the cells with 40 nM T2 toxin had less effects on the amino acid contents but resulted in an accumulation of asparagine (0.8-fold), aspartate (0.7-fold) and argininosuccinate (0.9-fold). In combination with the decrease of ornithine (-0.8-fold), urea (-0.2-fold) and *N*-acetylglutamate (-7.1-fold), an influence of T2 on the urea cycle was demonstrated (Fig. 3c). It has been described previously that T2 increases the activity of inducible NO synthase in rat pituitary gland tumor cells (GH3 cells) and therefore increases the intracellular NO concentration (Liu et al. 2017). An enrichment of NO could be involved in effects on the urea cycle as arginine, which is an intermediate of the urea cycle, serves also as a substrate for the NO synthase. The radical nature of NO can additionally induce the dimerization of glutathione (GSH), which may cause the increased concentration of glutathione disulfide (GSSG, 0.5-fold/0.8-fold) in the cell extract. T2 has been described to induce the expression of genes encoding antioxidant enzymes such as superoxide dismutase, glutathione reductase as well as glutathione peroxidase in mice (Chaudhary and Rao 2010). An inhibition of these enzymes may also increase the formation of GSSG. Additionally, a significant depletion of DNA building blocks (CMP, -0.4-fold, CDP, -0.6-fold, CTP, -0.5-fold, uridine diphosphate (UDP), -0.9-fold) and cellular DNA (Figure S3) was observed after treatment with 40 nM and indicative of an inhibition of DNA synthesis, also reported previously (Thompson and Wannemacher 1990; Rosenstein and Lafarge-Frayssinet 1983). In addition, the decrease of NADP^+^ (-0.6-fold), flavin adenine dinucleotide (FAD, -0.4-fold) and NaMN (-1.0-fold) as well as the decrease of several nucleotides suggest an inhibition of the PPP, which is in line with the decreased levels of sedoheptulose 7-phosphate (S7P, -0.7-fold) and pentose-P (-0.8-fold). Further effects include lower concentrations of many glycolysis metabolites (3-PG (-1.3-fold), hexose-P (-0.7-fold), glycerol-3P (-0.6-fold), PEP (-0.5-fold)) and an enrichment of UDPGA (1.5-fold), especially after 20 nM treatment with T2 toxin. UDPGA is a metabolite required for glucuronidation and detoxification of T2 toxin and the generation of T2 glucuronide has previously been reported in human cells (Welsch and Humpf 2012; Weidner et al. 2012). In general, T2 affects various metabolic pathways in a concentration-dependent manner.

### Deoxynivalenol

Treatment of cells with deoxynivalenol (DON) at concentrations of 100 nM and 200 nM caused a significant depletion of triose-P (-0.9-fold/-0.9-fold) as well as glycerol-3P (-0.6-fold/-0.5-fold, Fig. 2d). An inhibition of the PPP fits to the reduction of S7P (-0.3-fold/-0.2-fold) and 6-phosphogluconate (6-PG, -0.3-fold/-0.3-fold) with a lower statistical significance. Additionally, an effect on the acetylated amino acids such as *N*-acetylglutamate (-1.2-fold) and *N*-acetylaspartate (-0.3-fold) was observed after incubation of 200 nM DON. The decrease of these analytes could be connected to an inhibition of the *N*-acetyl amino acids synthase, as also suggested previously based on metabolic profiling of mice urine after DON treatment (Ji et al. 2018). Additional significantly increased metabolites were inosine triphosphate (ITP, 0.1-fold) and dihydrobiopterin (1.4-fold), whereas betaine (-2.2-fold) was decreased. Although DON and T2 have the same trichothecene backbone, they affected completely different metabolic pathways in HepG2 cells. This demonstrates that even small variations in chemical structures can be crucial in their toxicological mode of action.

### Metabolic effects caused by *Penicillium* and *Aspergillus* mycotoxins

32 different mycotoxins have been described as secondary metabolites of *Penicillium* species. The structural diversity ranges from small molecules like patulin (PAT) and citrinin (CIT) to more complex ochratoxins, penitrems and secalonic acids (Otero et al. 2020). *Aspergillus* species also produce a variety of secondary metabolites with aflatoxins and ochratoxin A (OTA) being the most important compounds (Perrone and Gallo 2017). The production of gliotoxin (GTX) and CIT is also described (Johannessen et al. 2007).

### Gliotoxin

GTX (100 nM and 200 nM) in the higher concentration induced a dimerization of GSH to GSSG (1.0-fold) and might influence the cellular redox state which has been previously suggested to be responsible for cell death (Kwon-Chung and Sugui, 2009). This effect could be related to the chemical structure of GTX (Figure S1) including a disulfide bridge which could interact with glutathione. In addition, the concentration of folate was reduced 0.4-fold (Fig. 2e). Folate is a precursor of the bioactive form tetrahydrofolate (THF), and both are very important in single carbon transfer reactions and synthesis of DNA building blocks. Slight, but significant increases of nucleotide monophosphates (guanosine monophosphate (GMP, 0.4-fold), adenosine monophosphate (AMP, 0.6-fold), CMP (0.2-fold), uridine monophosphate (UMP, 0.3-fold)) and the reduction of nucleosides adenosine (-0.3-fold), cytidine (-0.5-fold), guanosine (-0.4-fold), uridine (-0.3-fold) were observed. In addition, GTX caused the depletion of various analytes of the glycolytic pathway like glucose (-0.5-fold), 3-PG (-0.3-fold), 1,3-BPG (-0.2-fold) and PEP (-0.2-fold) in the cell extract. The citric acid cycle was not affected but a slight influence on the urea cycle was observed as arginine (-0.3-fold) and aspartate (-0.2-fold) were reduced and the contents of *N*-acetylglutamate (0.4-fold) and asparagine (0.6-fold) were increased. A significant enrichment of betaine (0.2-fold) and proline (0.5-fold) was also seen.

In previous publications, a modulation of the cellular redox state has been suggested to be responsible for the GTX-induced cell death (Kwon-Chung and Sugui 2009). Nevertheless, the metabolic profiling analysis conducted in this study revealed an interference with nucleotide synthesis, glycolysis and urea cycle which could also be expected to affect cell viability.

### Penitrem A

Penitrem A (Pen A, 10 µM and 25 µM) induced a concentration-dependent accumulation of different proteinogenic amino acids (Fig. 2f). These include arginine (0.1-fold/0.4-fold), asparagine (1.3-fold/1.9-fold), aspartate (0.1-fold/0.3-fold), glutamine (0.2-fold/0.5-fold), glutamate (0.1-fold/0.3-fold), histidine (0.2-fold/0.4-fold), isoleucine (0.4-fold/0.6-fold), leucine (0.1-fold/0.3-fold), lysine (0.1-fold/0.5-fold), methionine (0.4-fold/0.6-fold), phenyl-alanine (0.2-fold/0.4-fold), serine (0.1-fold/0.4-fold), threonine (0.2-fold/0.5-fold), tryptophan (0.2-fold/0.5-fold) and valine (0.2-fold/0.5-fold), which suggests a general inhibition of the protein biosynthesis. Norris *et al*. have previously reported that Pen A increases the release of glutamate, aspartate and γ-aminobutyrate (GABA) in cortical synaptosomes (Norris et al. 1980). The observed enrichment of the second messenger and neurotransmitter acetylcholine (0.8-fold/1.1-fold) in addition to the transmitter amino acids underline previous publications as tremorgenic effects are often attri-buted to Pen A (Arp and Richard 1981). According to the presented results of metabolic profiling Pen A doesn’t only influence neurotransmitter biosynthesis but also enhances argininosuccinate (0.8-fold/1.1-fold) and urea (0.2-fold/0.4-fold) suggesting an impact on the urea cycle (Fig. 3d).

In addition to the effect on the amino acid homeostasis, Pen A significantly affected key elements of the glycolytic pathway. Several analytes like G6P (0.2-fold/0.4-fold), F6P (0.5-fold/1.1-fold) and glucose 1-phosphate (G1P, 0.2-fold/0.5-fold) as well as glycerol-3P (0.5-fold/0.6-fold) were significantly enriched depending on the selected concentration of Pen A. UDP-glucose (0.6-fold/1.5-fold), which is important for the gluconeogenesis as well as UDPGA (1.3-fold/2.7-fold) as cofactor for glucuronidation were also accumulated. Concerning the PPP, an increase of 6-PG (0.1-fold/0.5-fold) and S7P (0.2-fold/0.6-fold) was observed, whereas a decrease of DNA building blocks such as inosine (-0.5-fold/-1.1-fold), cytidine (-0.4-fold/-0.9-fold), and adenosine (-0.5-fold/-0.8-fold) as well as NaMN (-1.4-fold/-0.8-fold) was shown.

A further important effect is the accumulation of GSSG (0.9-fold/2.5-fold) by Pen A which might be related to the generation of reactive oxygen species (ROS) as the ratio of GSH and GSSG has also been applied to evaluate the induction of oxidative stress previously (Rahman et al. 2006). Berntsen *et al*. have described the generation of ROS in human neutrophils at low micromolar concentrations of Pen A using the dichlorodihydrofluorescein fluorescence assay. An increase in intracellular calcium concentration and an activation of the MAPK signaling pathways have been supposed as critical mechanisms responsible for ROS formation (Berntsen et al. 2017). Betaine was also enriched significantly (0.3-fold/0.5-fold) but not further considered.

### Auranthine

The main effect for auranthine (AUR, 50 µM and 100 µM, Figure S6) was the reduction of several nucleosides like adenosine (-0.4-fold), guanosine (-0.7-fold), inosine (-0.8-fold), cytidine (-0.7-fold) and uridine (-0.6-fold) being involved in DNA and RNA synthesis by 100 µM AUR. The accumulation of nucleotide triphosphates such as adenosine triphosphate (ATP, 0.2-fold), ITP (0.2-fold) and CTP (0.4-fold) demonstrate an effect on cellular energy metabolism. The slight enrichment of UDP-glucose (0.3-fold), F6P (0.3-fold) and glycerol-3P (0.3-fold) as well as the decrease of *myo*-inositol (-0.2-fold) might be related to an influence on glucose metabolism. The content of 6-PG (0.2-fold) was also slightly but significantly enlarged. The enrichment of UDPGA (0.7-fold) as a cofactor of glucuronidation is assumed to be involved in the metabolization of AUR. Additionally, 100 µM AUR like Pen A also increased the concentration of GSSG (1.0-fold) and ornithine (0.2-fold). Despite the number of cellular alterations, no specific mode of action was deduced.

### Patulin

PAT at concentrations of 1 µM and 2 µM did not cause strong metabolic alterations in HepG2 cells after 24 h of treatment. Only some analytes such as nucleotides or oxoglutarate were slightly enhanced but without high statistical significance and were not further considered. 1,3-BPG (0.2-fold) was the only significantly increased analyte. One explanation might be that patulin is reacting rather quickly with SH-groups of cysteine in cell culture medium as it has also been described to form various adducts with *N*-acetylcysteine or GSH (Fliege and Metzler 2000).

### Citrinin

CIT was tested at two concentrations, 20 and 40 µM, respectively. This compound affected the citric acid cycle as oxoglutarate (1.7-fold/1.3-fold) and coenzyme A (CoA, 4.2-fold/3.7-fold) were enriched, whereas acetyl-CoA (-0.1-fold/-0.8-fold) was decreased (Fig. 2g). These results were similar to those of MON which seemed to induce an inhibition of the oxoglutarate dehydrogenase and the pyruvate dehydrogenase. In published data on the impact of CIT on mitochondrial proteins, both an inhibition of the calcium (Ca) uptake in mitochondria and an inhibition of Ca-dependent enzymes have been described (Chagas et al. 1995) (Fig. 3e). The pyruvate dehydrogenase is a Ca-dependent enzyme that incorporates pyruvate into the citric acid cycle and catalyzes the reaction of CoA to acetyl-CoA. NAD^+^ serves a cofactor in this reaction and thereby is reduced to NADH which fits to an increase of NAD^+^ (0.4-fold/0.2-fold). The metabolization of oxoglutarate to succinyl-CoA as a second Ca-dependent enzymatic reaction is catalyzed by oxoglutarate dehydrogenase which metabolizes NAD^+^ to NADH as well. The inhibition of this reaction might be involved in the enrichment of oxoglutarate. Additionally, the concentration of lactate decreased slightly (-0.2-fold, 40 µM) which could be a result of less energy generation via citric acid cycle, so that lactate is oxidized to pyruvate (0.7-fold, 40 µM), which could be further used for gluconeogenesis.

The content of dihydrobiopterin was also increased (1.3-fold/1.1-fold) similar to the effect induced by DON but with lower significance (p ≤ 0.01). The pterin biosynthetic pathway as well as tetrahydrobiopterin-dependent enzymes could be affected, as the formation of tetrahydrobiopterin is associated with the level of dihydrobiopterin. As only one metabolite of the pterin biosynthetic pathway was present in sufficient concentrations in HepG2 cells, no reliable conclusions could be drawn about this metabolic effect.

An additional effect was the significant decrease of glycerol-3P (-0.5-fold/-0.6-fold) and triose-P (-0.1-fold/-0.5-fold), which are metabolites of the glycerol 3-phosphate dehydrogenase. This enzyme works also in a Ca-dependent manner thus a reduced Ca level is assumed to affect this reaction (Mráček et al. 2013). The reduced concentration of triose-P might be directly connected to the decrease of S7P (-0.3-fold/-0.7-fold) concentration being involved in the PPP. The not specified hexose phosphate (1.1-fold/0.6-fold), aspartate (0.2-fold/0.1-fold), and UDPGA (0.8-fold/0.7-fold) were also significantly increased and *N*-acetylglutamate (-1.6-fold/-1.7-fold) as well as niacinamide (-0.3-fold/-0.3-fold) were reduced by CIT.

### Aflatoxin B_1_

Aflatoxin B_1_ (AfB_1_, 10 µM and 20 µM) had significant effects on the metabolome especially after testing of 20 µM as many analytes were downregulated by this concentration (Fig. 2h). Intermediates of the PPP such as S7P (-0.8-fold) and 6-PG (-0.8-fold) were reduced as well as NADH (-0.6-fold) and NADP^+^ (-0.6-fold). These results might be related to an inhibited DNA synthesis which has already been reported previously in mammalian cells after AfB_1_ treatment (Meneghini and Schumacher 1977; Ricordy et al. 2002). The reduced DNA content determined via GelGreen fits also to these results (Online Resource 1, Figure S3).

The accumulation of UDPGA (0.4-fold) and a contrasting downregulation of UDP-glucose (-0.8-fold), glucose (-0.5-fold) and PEP (-0.7-fold) by 20 µM AfB_1_ are in line with previous in vivo studies in rats. The results could be related to a higher cellular glucose utilization caused by AfB_1_ and a direct influence on the gluconeogenesis (Lu et al. 2013). Further influenced metabolites include methenyl-THF (1.7-fold, 20 µM) and creatine (-0.5-fold, 20 µM). Additionally, many amino acids were reduced particularly serine (-0.4-fold, 20 µM) and aspartate (-0.8-fold, 20 µM).

### Ochratoxin A

The treatment of HepG2 cells with 200 nM and 400 nM OTA influenced the glycolytic pathway, which is important for the energy supply of the cells (Fig. 2i, Fig. 3f). The hexose phosphates like G1P (-0.8-fold), G6P (-0.4-fold) and F6P (-0.6-fold) as well as the not specified hexose phosphate (-1.0-fold) were depleted in the cell extract which indicates the inhibition of glucose metabolism by 400 nM OTA. It has been previously reported by Hundhausen et al. that the protein levels of some enzymes of the carbohydrate metabolism were reduced by OTA in HepG2 cells. These include for example the key enzyme phosphofructokinase catalyzing the conversion of F6P to fructose 1,6-bisphosphate (F-1,6-BP). The content of phosphoglycerate kinase which is responsible for the conversion of 1,3-BPG to 3-PG was also reduced in this study, (Hundhausen et al. 2008). These variations in enzyme expression correlate well with the above-mentioned results of metabolic profiling as many compounds involved in glycolysis were decreased [3-PG (-1.3-fold), F6P, G1P, G6P, hexose-P, and PEP (-0.5-fold)].

The alterations of the glycolysis are associated with a modulation of the urea and the citric acid cycle, respectively, as key elements of those pathways such as fumarate (0.5-fold) and aspartate (-0.3-fold) are slightly affected at higher OTA concentrations. Especially, the concentration of *N*-acetylglutamate (6.1-fold) was reduced by OTA.

Metabolites of the PPP, like S7P (-1.7-fold), 6-PG (-0.6-fold), pentose-P (-0.8-fold), which are intermediates of the synthesis of DNA and RNA building blocks were also downregulated by 400 nM OTA, although no decrease of nucleoside concentrations was observed. The amino acids involved in the neurotransmitter synthesis like tyrosine (-0.9-fold), glutamate (-0.3-fold), and tryptophan (-0.3-fold) as well as the cyclic adenosine monophosphate (cAMP) as a second messenger (-2.1-fold) were significantly decreased; thus, an impaired signal transduction is suggested. Further amino acids, such as proline (-1.7-fold) and aspartate (-0.3-fold) as well as the nucleosides cytosine (-1.2-fold) and orotidine (-1.3-fold), were found in lower concentrations as well as betaine (-0.3-fold).

### Metabolic effects caused by *Stachybotrys* mycotoxins

*Stachybotrys chartarum* is a fungal species growing on indoor materials such as wallpaper, especially in water-damaged buildings. Macrocyclic trichothecenes such as satratoxins as well as phenylspirodrimanes belong to the secondary metabolites of this fungal species (Islam et al. 2009; Jarvis et al. 1995; Jagels et al. 2019).

**Satratoxin G** (Sat G) (2 nM and 4 nM) reduced the content of DNA and RNA building blocks, which might be related to an influence on the synthesis of DNA and RNA which was also observed in GelGreen-Assay (Online-Resource 1, Figure S3). These results are in line with the significant reduction of S7P (-0.3-fold/-0.2-fold) and 6-PG (-0.4-fold/-0.4-fold) as metabolites of the PPP (Fig. 2j). Additionally, some analytes of the glycolysis and the citric acid cycle were depleted for example malate (-0.2-fold/-0.2-fold), succinate (-0.6-fold/-0.4-fold), PEP (-0.5-fold/-0.3-fold) and 3-PG (-0.4-fold/-0.5-fold) which implicates an effect on cellular energy metabolism. The concentrations of different amino acids such as isoleucine (-0.2-fold/-0.2-fold), glutamine (-0.3-fold/-0.3-fold), phenylalanine (-0.1-fold/-0.2-fold), and asparagine (-0.9-fold/-1.0-fold) were reduced whereby asparagine showed the strongest decrease. Although many metabolites were downregulated by the test compound, a specific mode of action was not deduced.

**Acetoxystachybotrydial acetate** (AcDialAc, 100 nM and 200 nM) induced similar effects as Sat G (Online-Resource 1, Figure S6). Asparagine was also the strongest affected amino acid (-0.7-fold/-1.1-fold), but further amino acids were also decreased. S7P (-0.4-fold/-0.3-fold), PEP (-0.4-fold/-0.1-fold) as well as different DNA and RNA building blocks such as uridine (-0.3-fold/-0.5-fold) and inosine (-0.3-fold/-0.6-fold) were also reduced.

### Metabolic effects caused by *Claviceps* mycotoxins

*Claviceps purpurea* is one of the fungi mostly observed in contaminated grains and other plants from Europe and it is responsible for the mycotoxicosis ergotism (Haarmann et al. 2009). Two important groups of *Claviceps* secondary metabolites are ergot alkaloids and ergot pigments such as secalonic acids (Neubauer et al. 2016).

### Secalonic acid A

At concentrations of 5 µM and 10 µM secalonic acid A (Sec A) induced a series of significant cellular effects. The amounts of succinate (-0.6-fold/-0.4-fold) were decreased by both Sec A concentrations and glucose (-0.6-fold) only by 5 µM Sec A (Online-Resource 1, Figure S6). The structurally related derivative secalonic acid D (Sec D) has been described to have cytotoxic effects on pancreatic epithelial carcinoma cells (PANC-1) under glucose deficient conditions but no cytotoxic potential in presence of glucose. An inhibition of Akt signaling survival pathway and an induction of the uncoupling effect at the mitochondrial membrane have also been reported. The expression of the glucose-related protein 78 was also reduced after treatment with 10 µM Sec D (Tang et al. 2020). These results lead to the assumption that cytotoxic effects of Sec A are related to the reduced cellular glucose concentrations. At the higher test concentration of 10 µM also a depletion of nucleosides, especially inosine (-0.9-fold), guanosine (-0.8-fold), uridine (-0.7-fold) and adenosine (-0.7-fold) were observed. Sec A seems to influence DNA synthesis and cell proliferation. Additionally, the content of creatine (-0.3-fold) was significantly decreased by 5 µM Sec A.

### Ergocristine

The ergot alkaloid ergocristine (ECR) was studied only at 50 µM according to previous experiments where ECR showed similar metabolic effects at higher concentrations. Because of only marginal cytotoxicity, the lower concentration of 50 µM was chosen. The concentrations of various nucleotides, especially nucleoside monophosphates such as AMP (0.7-fold), CMP (-1.1-fold), GMP (-1.1-fold) and UMP (-0.7-fold) as well as CTP (-3.0-fold) and GDP (-0.4-fold) were significantly reduced, which is a hint to an interruption of the energy metabolism (Fig. 2k). Additionally, cytosine (-3.7-fold) and guanosine (-1.3-fold) as nucleosides were decreased and the overall DNA concentration was reduced (Online-Resource 1, Figure S3), although 6-PG (0.1-fold, not significant) and S7P (0.3-fold) levels were only little modulated by ECR. Several metabolites of the urea cycle such as *N*-acetylglutamate (1.4-fold), argininosuccinate (0.8-fold) and asparagine (0.7-fold) were increased, while aspartate was decreased 1.1-fold (Fig. 2k). The impact on elements of the citric acid cycle, except the enrichment of succinate (0.7-fold), are comparable to those of ZEN, which also enhanced the amount of oxoglutarate (0.6-fold) and decreased the substrates fumarate (-0.3-fold), pyruvate (-1.2-fold) and CoA (-5.0-fold). In contrast, the glycolysis was only marginally affected. G6P was the only enriched analyte (0.6-fold) and pyruvate and *myo*-inositol (-1.8-fold) were significantly reduced.

Furthermore, a generation of ROS by ECR is suggested, because incubation experiments led to an accumulation of GSSG (0.9-fold). In literature, an effect on neurotransmission has been reported (Klotz 2015), which might be related to our findings which show a depletion of serine (-0.4-fold) and glutamate (-0.3-fold) as well as an enrichment of tyrosine (0.7-fold) and tryptophan (0.4-fold) as neurotransmitter precursor. Additional enriched analytes were histidine (0.3-fold) and betaine (1.4-fold). In contrast, NADP^+^ (-0.6-fold) and sphingosine (-3.5-fold) were decreased.

### Metabolic effects caused by *Alternaria* mycotoxins

All of the assayed A*lternaria* toxins induced just minor effects on the metabolome of HepG2 cells. An impact of **alternariol** (AOH, 1 µM and 10 µM) on the intracellular redox status is expected, because a slight increase of GSSG (0.6-fold/0.8-fold) was found. Additionally, the concentrations of tyrosine (-0.3-fold/-0.4-fold) and tryptophan (-0.2-fold/-0.2-fold) as precursor compounds of several neurotransmitters were decreased by AOH; thus, an effect on the signal transduction can be supposed. Pentose-P (-0.6-fold/-0.1-fold) and betaine (-0.2-fold/-0.3-fold) were decreased significantly (Figure S6).

A comparable increase of GSSG (0.4-fold/0.9-fold) was also observed for **altertoxin I** (ATX I, 1 µM and 10 µM) which may be due to its structural similarity with alternariol. Furthermore, 10 µM ATX I induced an enrichment of F6P (0.4-fold), G6P (0.2-fold) and G1P (0.4-fold) and a decrease of *N*-acetylglutamate (-0.3-fold, Figure S6). As 10 µM ATX I did not induce cytotoxic effects in the resazurin assay a higher concentration might lead to stronger metabolic effects.

**Tenuazonic acid** (TeA, 10 µM and 100 µM) in the higher tested concentration diminished the amounts of the amino acids tyrosine (-0.6-fold), threonine (-0.3-fold), asparagine (-0.7-fold) and aspartate (0.1-fold) which might be related to an impaired protein biosynthesis (Online-Resource 1, Figure S6). TeA also influenced glycolytic compounds such as F6P (0.7-fold), G6P (0.4-fold) and UDP-glucose (0.5-fold). Ornithine (-0.5-fold) and creatine (-0.3-fold) are further metabolites decreased significantly by 100 µM TeA. In contrast, GSSG was significantly accumulated (0.3-fold).

**Altenuene** (ALT, 1 µM and 10 µM) significantly decreased the concentration of glucose (-0.3-fold/-0.4-fold) and consequently seemed to influence the energy metabolism of the cells (Online-Resource 1, Figure S6). The decrease of nucleosides such as inosine (-0.7-fold), guanosine (-0.6-fold), uridine (-0.5-fold) and adenosine (-0.5-fold) reached only low significance.

**Fig. 4:**
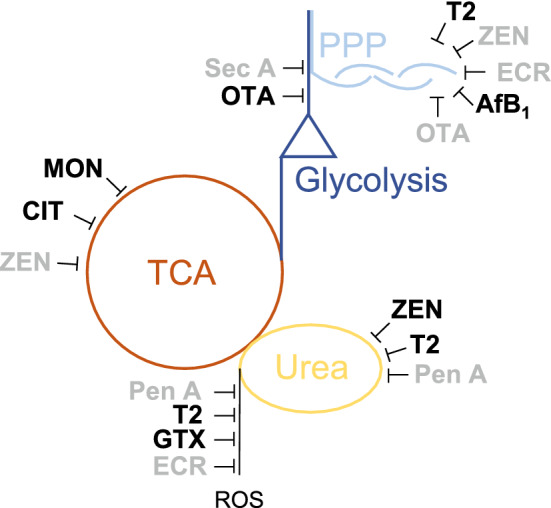
Graphical summary of suggested metabolic effects caused by selected mycotoxins. Effects which are consistent with literature data are marked in black and effects which could not be assigned to literature are colored in grey. *ROS* reactive oxygen species, *PPP* pentose phosphate pathway, *TCA* citric acid cycle

The results of this study clearly show that certain mycotoxins exert specific effects on metabolic pathways as summarized in Fig. 4. As one of the most striking effects of the investigated mycotoxins, the amino acid homeostasis was influenced by Pen A as well as T2 in a dose-dependent manner. The citric acid cycle was especially altered by MON, ZEN and CIT, which also influenced the glycolysis and the energy metabolism. OTA and Sec A also seemed to affect the glycolysis, while ECR, FB_1_ and ZEN had an effect on the sphingosine metabolism. The PPP as well as the DNA and RNA synthesis were affected by several mycotoxins, but especially the *Fusarium* toxins were characterized as potent inhibitors of several analytes involved in these pathways. The urea cycle was altered by ZEN, T2 and Pen A, and an effect on the generation of ROS was suggested especially for Pen A, T2, GTX and ECR because of a higher GSSG concentration. For some mycotoxins such as T2 or FB_1_ specific analytes and metabolic pathways were affected stronger by the lower tested concentration which might be due to the close interrelation of metabolic pathways. Different mycotoxin concentrations might influence the same metabolic pathways at different reaction steps inducing secondary or reverse effects at higher mycotoxin concentrations because of overlapping metabolic reactions. To cover these effects, the mycotoxins were tested at two different concentrations.

## Conclusion

The study demonstrates that most of the studied mycotoxins strongly interfere with different metabolic pathways in HepG2 cells. In some cases, the observed effects on the cellular metabolome confirm the existing literature data explaining the mode of action of the studied mycotoxins. In several other cases, the data reveal potentially novel toxicological endpoints and might be relevant for further risk eva-luation. Notably, most metabolic alterations were observed at mycotoxin concentrations that did not impair cellular viability. These results indicate that metabolic alterations do not inevitably cause cell death, but make metabolic profiling a more sensitive method to discover toxicological endpoints. For example, MON did not exhibit cytotoxic effects up to 100 µM, but an influence on the metabolome of HepG2 cells was already observed very clearly after treatment with 50 µM. As it has been reported previously, mycotoxins induce cytotoxic effects in vivo depending on the target organ. Therefore, the limitation of the present study on one hepatocarcinogenic cell line needs to be considered. The extension of the study on further cell lines might be helpful and complete the data for some mycotoxins such as OTA, which is described to primarily affect kidney cells (Gekle et al 2005). HepG2 cells were chosen as the liver is responsible for the metabolization of xenobiotics and hepatocytes are exposed to high concentration of those, which makes the investigation of effects induced by xenobiotics especially re-levant. In addition, the differences between cancer cell lines and primary cells as well as the in vivo situation need to be considered, as cancer cell lines frequently exhibit reduced metabolic activity and could not entirely reflect the in vivo situation. Nevertheless, the use of cancer cell lines is suitable for metabolic profiling as lower biological variation allows a more reliable interpretation of complex data.

Taken together, the developed metabolic profiling method provided detailed insight in cellular responses to mycotoxin exposure enabling more focused studies of their molecular effects in vivo and in vitro. Moreover, the method can be applied to study the bioactivity of novel secondary metabolites of various origin accelerating research regarding their effects on human cells.

## Supplementary Information

Below is the link to the electronic supplementary material.

Supplementary file1 (XLSX 212 KB)

Supplementary file2 (DOCX 7138 KB)

## Declarations

### Data Availability

All relevant datasets of the current study are available in the published article or in Online Resources 1 and 2.
