# Supplementary material for: Metabolic profiling as a powerful tool for the analysis of cellular alterations caused by 20 mycotoxins in HepG2 cells
Source: Arch Toxicol. 2022 Aug 6;96(11):2983–98. doi: 10.1007/s00204-022-03348-5 (PMC9525358; doi:10.1007/s00204-022-03348-5)
Supplement: Supplementary file 2 — Supplementary file2 (DOCX 7138 KB) [file 204_2022_3348_MOESM2_ESM.docx]

**Supplementary material**

**for**

**Metabolic profiling as a powerful tool for the analysis of cellular alterations caused by 20 mycotoxins in HepG2 cells**

Andrea Gerdemann^1^ (andrea.gerdemann@wwu.de), Matthias Behrens^1^, Melanie Esselen^1^, Hans-Ulrich Humpf^1^, ^*^ (humpf@wwu.de)

^1^Institute of Food Chemistry, University of Münster, Corrensstraße 45, 48149 Münster, Germany

**Corresponding Author:**

*Prof. Dr. Hans-Ulrich Humpf, Institute of Food Chemistry, 48149 Münster, Germany; e-mail address: humpf@wwu.de

Journal: Archives of Toxicology

**Fig. S1** Chemical structures of tested mycotoxins

**Preparation of incubation solutions**

Stock solutions of the mycotoxins were prepared either in dimethylsulfoxide (DMSO) or in acetonitrile (ACN) depending on their stability and solubility. Moniliformin (MON), fumonisin B_1_ (FB_1_), satratoxin G (Sat G) and acetoxystachybotrydial acetate (AcDialAc) were dissolved in DMSO and the remaining mycotoxins were dissolved in ACN.

**Tab. S1** Purities and concentration of incubated mycotoxins

| **Substance** | **Purity** | **Determination** | **Incubated concentration** | **Source** |
| --- | --- | --- | --- | --- |
| Moniliformin | > 95 % | LC-UV (260nm) | 5 µM/50 µM | (Lohrey et al. 2011) |
| Fumonisin B_1_ | > 98 % | HPLC-ELSD | 10 µM/100 µM | (Hübner et al. 2012) |
| T2 toxin | > 90 % | ^1^H NMR | 20 nM/40 nM | (Beyer et al. 2009) |
| Deoxynivalenol | ≥ 95 % | - | 100 nM/200 nM | (Bretz et al. 2006) |
| Zearalenone | ≥ 98 % | - | 25 µM/50 µM | Fermentek, Jerusalem, Israel |
| Penitrem A | 99 % | HPLC-DAD-ELSD | 10 µM/25 µM | (Kalinina et al. 2017) |
| Auranthine | 97 % | HPLC-DAD-ELSD | 50 µM/100 µM | (Kalinina et al. 2018) |
| Patulin | Recrystallized from diethyl ether | | 1 µM/2 µM | isolated in-house |
| Gliotoxin | ≥ 98 % | TLC | 100 nM/200 nM | VWR, Darmstadt, Germany |
| Ochratoxin A | > 99 % | HPLC-UV | 200 nM/400 nM | (Sueck et al. 2019) |
| Citrinin | > 98 % | - | 20 µM/40 µM | biomol, Hamburg, Germany |
| Aflatoxin B_1_ | ≥ 98 % | - | 10 µM/20 µM | Acros organics, Geel, Belgium |
| Acetoxy-stachybotrydial acetate | 95 % | HPLC-DAD-ELSD | 100 nM/200 nM | (Jagels et al. 2019) |
| Satratoxin G | 97 % | HPLC-DAD-ELSD | 2 nM/4 nM | (Jagels et al. 2019) |
| Secalonic acid A | 96 % | HPLC-DAD-ELSD | 5 µM/10 µM | (Lünne et al. 2021) |
| Ergocristine | - | - | 50 µM | E140, Sigma Aldrich, Steinheim, Germany |
| Tenuazonic acid | > 95 % | - | 10 µM/100 µM | (Hickert et al. 2016) |
| Altertoxin I | > 97 % | HPLC-ELSD | 1 µM/10 µM | (Hickert et al. 2016) |
| Alternariol | > 95 % | HPLC-ELSD | 1 µM/10 µM | (Hickert et al. 2016) |
| Altenuene | > 98 % | HPLC-ELSD | 1 µM/10 µM | (Hickert et al. 2016) |

**Tab. S2** Analytes included in multi-standard solution at a concentration of 10 µg/mL

| **Compound** |
| --- |
| Glucose 1-phosphate |
| Fructose 1,6-phosphate |
| 3-Phosphoglycerate |
| Dihyroxyacetone phosphate |
| Glucosamine |
| NADH |
| UDPGA |
| Creatine |
| Fumarate |
| Succinate |
| Aspartate |
| Acetyl-CoA |
| GSH |
| Nicotinate |
| *N*-acetylglutamate |
| Serotonine |
| Citrulline |
| Glucose |
| Uric acid |
| Adipic acid |
| Biotin |
| NADPH |
| Choline |
| Glucuronic acid |
| Cysteine |
| Pyruvate |
| Fructose |
| *myo*-inositol |
| Ornithine |
| Hypoxanthine |
| Pyridoxal phosphate |
| Nicotinamide |
| Creatinine |
| Urea |
| Dihydrobiopterin |
| Dihydrofolate |
| Levomefolate |
| Biopterin |
| Coenzyme A |

**Cytotoxicity Assay**

Resazurin reduction assay was performed according to a previous publication (O’Brien *et al*. 2000) 15 000 HepG2 cells/well were seeded in a 96 well plate. After 24 h the medium was replaced by serumfree medium and 48 h after seeding the cells were incubated with the specific mycotoxins. The stock solutions were diluted by 100 to ensure a maximum solvent concentration of 1 %. For each mycotoxin at least four different concentrations were tested. Additionally, a negative control of 1 % of ACN or DMSO in cell culture medium (according to the used solvent of the mycotoxin) as well as a positive control of 10 µM T2 toxin were included in each experiment. After 24 h of incubation 10 µL resazurin (440 µM) were added and incubated 120 min at 37 °C. The metabolic activity was measured at 544 nm excitation and 590 nm emission using a microplate reader (Tecan, Gröding, Austria). The cytotoxicity was calculated relative to the solvent control. As the cytotoxicity of the substances just served as an orientation for chosen sub-toxic concentrations for metabolic profiling the experiment was only performed in *n*= 1 × 6. The results of cytotoxicity assay are shown in Figure S2.

**Fig. S2** Determination of cytotoxicity of all compounds on HepG2 cells after 24 h of incubation using resazurin reduction assay. The assay was performed to estimate sub-toxic concentrations used for metabolic profiling (*n*= 1 × 6). 10 µM of T2 toxin was used as positive control in all experiments. The values are calculated relative to solvent control and statistical significance is shown according to the results of Student´s T-test (***: p ≤ 0.001, **: p ≤ 0.01, *: p ≤ 0.05).

**DNA quantification using GelGreen-Assay**

The DNA concentration was calculated using a DNA intercalating dye to get an estimation on the cell count. First the cell pellet was resuspended in 100 µL DNase free water and sonicated 15 min. After centrifugation (15 min, 4 °C, 14840 × g) the supernatant was diluted by 20 with sterile TE buffer (10 mM Tris, 1 mM EDTA) and used for DNA quantification. The DNA quantification was performed in black and sterile 96 well plates (Thermo, Bremen, Germany). 5 µL of the diluted sample were further diluted with 95 µL of a mastermix. For the mastermix 1 µL of 100x GelGreen (Merck Millipore, Darmstadt, Deutschland) was mixed with 94 µL of buffer per sample. The mastermix was prepared freshly before every set of quantification. A calibration curve ranging from 156.3 ng/mL to 10.0 µg/mL was also added to the dataset as well as a cell pellet generated from different cell counts to get an estimation on the cell count of the samples. The data are shown in Figure S5 as fold change to the solvent control based on the DNA concentration.

The incubation of ZEN, T2, OTA, ECR, AOH, AfB_1_ and Pen A reduced the DNA content significantly. This effect might be caused by cytotoxic effects and a reduced cell count. Additionally, an inhibited DNA synthesis might be the reason for reduced DNA concentrations. In contrast the DNA concentration after incubation of DON was increased significantly which might be due to an increased DNA synthesis or a higher cell count

**Fig. S3** Determination of the DNA/RNA content using DNA intercalating dye GelGreen shown as fold change relative to solvent controls. GelGreen assay was used to estimate the cell count. As AfB_1_ was incubated separately, a separate solvent control was used. The values are calculated as fold change in comparison to solvent control including the respective significance levels *** p ≤ 0.001, ** p ≤ 0.01, * p ≤ 0.05 according to Student´s T-test (*n* = 3 × 2).

**Chromatographic and mass spectrometric parameters**

Before each sample set the HILIC‑Z column was flushed with 20 % of solvent A for 30 min (0.5 mL/min) to remove remaining contaminants of previous measurements. Afterwards a 30 min equilibration period using 85 % of solvent A was used to prepare the column for the measurement. The gradient started with 2 min of 85 % A followed by a decrease to 20 % in 5 min. After 1 min, solvent A was increased to 85 % in 0.1 min and kept constant for 6.9 min. In the equilibration period the flow rate was increased to 0.7 mL/min for 5.4 min in total to obtain an equilibration volume of 12 column volumes, which has been described to generate reproducible results (McCalley 2020). The column oven was adjusted to 40 °C.

The source parameters of the EVOQ mass spectrometer were adjusted to the flow rate of 0.5 mL/min. Therefore, spray voltages of 5000 V in positive mode and 4500 V in negative mode were selected. The cone temperature was adjusted to 250 °C and the Cone Gas Flow was 20. The heated probe had a temperature of 500 °C and the Probe Gas Flow and the Nebulizer Gas Flow were adjusted to 50 and 60 respectively. The Exhaust gas was also activated. The detailed parameters for each transition are listed in the Online Resource 2.

**LC-MS method development**

Important analytes in metabolic profiling are small and polar molecules like amino acids, organic acids or nucleotides. Hydrophilic interaction chromatography (HILIC) is a useful tool for the chromatographic separation of this kind of molecules as it combines the advantages of normal phase chromatography, reversed phase chromatography and ion chromatography.

For method development and optimization of the chromatographic conditions a HPLC‑HRMS (high performance liquid chromatography - high resolution mass spectrometry) combination using time-of-flight mass spectrometer (Impact II, Bruker Daltonics, Bremen, Germany) was used. A mixture of different cell extracts (reference cell extract) served as a sample, which allowed the inclusion of matrix effects of a real sample directly in method development and included a huge variety of analytes. In a first step the influence of stainless-steel components on the chromatography was investigated. Therefore, the stainless-steel capillary between the autosampler valve and the separation column with a length of approximately 1 m was replaced by a PEEK (polyetheretherketone) capillary. Less tailing and reduced noise were achieved for example for organic acids like lactic acid or sugar phosphates (Figure S3). In previous publications it has been mentioned that organic phosphates interact with stainless-steel components of the system. The short-term absorption leads to tailing and asymmetric chromatographic peaks. The use of shorter stainless-steel capillaries or a replacement of those components reduced this effect (Tuytten et al. 2006; Shi et al. 2002).

The stationary phase of HPLC columns has a strong influence on the chromatographic results. Zwitterionic HILIC phases (HILIC‑Z) include positively and negatively charged end groups and they are suitable for very polar and charged analytes. The replacement of a HILIC column by a HILIC‑Z column significantly improved the chromatographic results for a variety of analytes. The peak shapes of organic acids and organic phosphates were improved significantly (Figure S3).

The HILIC‑Z column was described to be stable at higher pH values. To gain a higher peak intensity due to better ionization conditions for anionic analytes the pH of the mobile phase was increased to pH 9. The modification of the pH had just slight effects on many analytes like a reduced retention time. As the higher pH had no significant negative effects even on positive charged analytes and was described to reduce the corrosion of stainless-steel components, pH 9 was used in following measurements. The improvement of the peak shape is shown in Figure S3. for selected analytes.

**Fig. S4** Optimization of chromatographic conditions using a HPLC-qToF combination. Improved peak shape of lactate, glutamate, phenylglycine and adenosine triphosphate (ATP) is illustrated. The replacement of a stainless-steel capillary with polyetheretherketone (PEEK) material and the use of a HILIC-Z column in comparison to a silica based HILIC column as well as a higher pH value improved the peak shape especially of organic acids and phosphates.

After chromatographic optimization and analyte annotation the method was transferred to a targeted approach using triple quadrupole mass spectrometer. An advantage of the targeted analysis is the higher sample throughput because of the simultaneous analysis in positive and negative ionization mode and the higher selectivity and sensitivity. The choice of the hardware was crucial, as the chromatographic results differed significantly.

A combination of a 1290 Infinity HPLC pump (Agilent, Waldbronn, Germany) and a QTRAP 5500 mass spectrometer (Sciex, Darmstadt, Germany) was compared to a coupling of an Elute pump (Bruker Daltonics, Bremen, Germany) and EVOQ mass spectrometer (Bruker Daltonics, Bremen, Germany). The Elute pump was the same HPLC pump also used for method development with the ToF mass spectrometer. The combination of the instruments provided by Bruker generated more symmetric peaks especially for phosphorylated analytes and organic acids and was well comparable to the HRMS measurement (Figure S4). The improved peak shape might be related to built-in stainless-steel components. Using the same HPLC pump for targeted and untargeted analysis offered an additional opportunity for analyte identification as retention times were more comparable and simplified the extension of the targeted method with new occurring analytes.


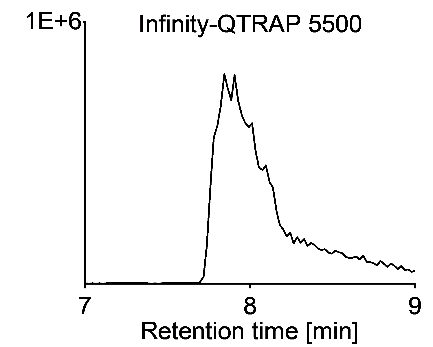

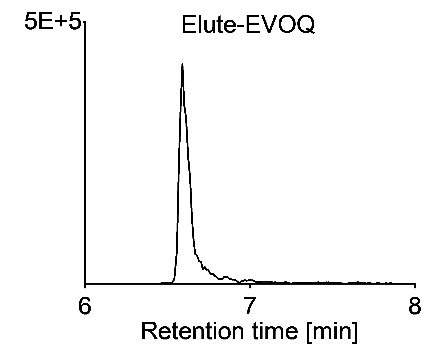

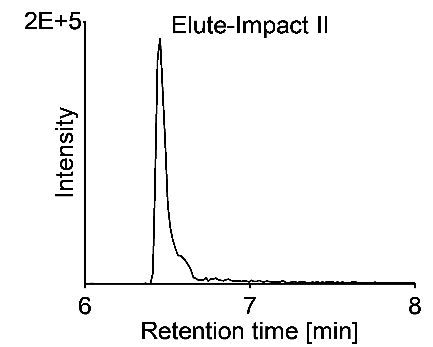


**Fig. S5** Comparison of chromatographic results of different combinations of HPLC pumps and mass spectrometer by the example of adenosine triphosphate in a cell extract. The use of an Elute pump generated more symmetric and narrower peaks than the Infinity 1290.

**Metabolic alterations caused by selected mycotoxins**

**Fig. S6** Selected metabolic alterations caused by remaining mycotoxins illustrated as bar graphs. The values are calculated as fold change in comparison to solvent control including the respective significance levels *** p ≤ 0.001, ** p ≤ 0.01, * p ≤ 0.05 according to Student´s T-test (*n* = 3 × 2). The color code assigns the analytes to different metabolic pathways and structural classes:  amino acids,  urea cycle, citric acid cycle,  glycolysis,  pentose phosphate pathway,  nucleoside derivatives

**Fig. S7** Principle component analysis of tested mycotoxins (a) including the loading plot (b). The coloration of the mycotoxins was chosen according to their producing fungi species. Blue shades: *Fusarium*, green shades: *Penicillium/Aspergillus*, purple shades: *Claviceps*, Yellow shades: *Stachybotrys*, red shades: *Alternaria*. The following analytes were excluded, because of missing values for aflatoxin B_1_: citrulline, CMP, triose phosphate, guanine, hypoxanthine, orotidine, pyruvate.

**b**

**a**

**References**

Beyer M, Ferse I, Mulac D, Würthwein E-U, Humpf H-U (2009) Structural elucidation of T-2 toxin thermal degradation products and investigations toward their occurrence in retail food. J. Agric. Food Chem. 57:1867–1875. <https://doi.org/10.1021/jf803516s>

Bretz M, Beyer M, Cramer B, Humpf H-U (2006) Stable isotope dilution analysis of the Fusarium mycotoxins deoxynivalenol and 3-acetyldeoxynivalenol. Mol. Nutr. Food Res. 50:251–260. <https://doi.org/10.1002/mnfr.200500230>

Cramer B, Bretz M, Humpf H-U (2007) Stable isotope dilution analysis of the fusarium mycotoxin zearalenone. J. Agric. Food Chem. 55:8353–8358. <https://doi.org/10.1021/jf0717283>

Hickert S, Bergmann M, Ersen S, Cramer B, Humpf H-U (2016) Survey of Alternaria toxin contamination in food from the German market, using a rapid HPLC-MS/MS approach. Mycotoxin Res 32:7–18. <https://doi.org/10.1007/s12550-015-0233-7>

Hübner F, Harrer H, Fraske A, Kneifel S, Humpf H-U (2012) Large scale purification of B-type fumonisins using centrifugal partition chromatography (CPC). Mycotoxin Res 28:37–43. <https://doi.org/10.1007/s12550-011-0114-7>

Jagels A, Lindemann V, Ulrich S, Gottschalk C, Cramer B, Hübner F, Gareis M, Humpf H-U (2019) Exploring Secondary Metabolite Profiles of Stachybotrys spp. by LC-MS/MS. Toxins 11. <https://doi.org/10.3390/toxins11030133>

Kalinina SA, Jagels A, Cramer B, Geisen R, Humpf H-U (2017) Influence of Environmental Factors on the Production of Penitrems A-F by Penicillium crustosum. Toxins 9. <https://doi.org/10.3390/toxins9070210>

Kalinina SA, Kalinin DV, Hövelmann Y, Daniliuc CG, Mück-Lichtenfeld C, Cramer B, Humpf H-U (2018) Auranthine, a Benzodiazepinone from Penicillium aurantiogriseum: Refined Structure, Absolute Configuration, and Cytotoxicity. J Nat Prod 81:2177–2186. <https://doi.org/10.1021/acs.jnatprod.8b00187>

Lohrey L, Murata T, Uemura D, Humpf H-U (2011) Synthesis of Isotopically Labeled Fusarium Mycotoxin ¹³C2-Moniliformin [1-Hydroxycyclobut-1-ene-3,4-dione]. Synlett 2011:2242–2244. <https://doi.org/10.1055/s-0030-1261189>

Lohrey L, Marschik S, Cramer B, Humpf H-U (2013) Large-scale synthesis of isotopically labeled 13C2-tenuazonic acid and development of a rapid HPLC-MS/MS method for the analysis of tenuazonic acid in tomato and pepper products. J. Agric. Food Chem. 61:114–120. <https://doi.org/10.1021/jf305138k>

Lünne F, Köhler J, Stroh C, Müller L, Daniliuc CG, Mück-Lichtenfeld C, Würthwein E-U, Esselen M, Humpf H-U, Kalinina SA (2021) Insights into Ergochromes of the Plant Pathogen Claviceps purpurea. J Nat Prod 84:2630–2643. <https://doi.org/10.1021/acs.jnatprod.1c00264>

McCalley DV (2020) Managing the column equilibration time in hydrophilic interaction chromatography. J Chromatogr A 1612:460655. <https://doi.org/10.1016/j.chroma.2019.460655>

O’Brien J, Wilson I, Orton T, Pognan F (2000) Investigation of the Alamar Blue (resazurin) fluorescent dye for the assessment of mammalian cell cytotoxicity. Eur J Biochem 267:5421–5426. <https://doi.org/10.1046/j.1432-1327.2000.01606.x>

Shi G, Wu J, Li Y, Geleziunas R, Gallagher K, Emm T, Olah T, Unger S (2002) Novel direct detection method for quantitative determination of intracellular nucleoside triphosphates using weak anion exchange liquid chromatography/tandem mass spectrometry. Rapid Commun Mass Spectrom 16:1092–1099. <https://doi.org/10.1002/rcm.684>

Sueck F, Hemp V, Specht J, Torres O, Cramer B, Humpf H-U (2019) Occurrence of the Ochratoxin A Degradation Product 2’R-Ochratoxin A in Coffee and Other Food: An Update. Toxins 11. <https://doi.org/10.3390/toxins11060329>

Tuytten R, Lemière F, Witters E, van Dongen W, Slegers H, Newton RP, van Onckelen H, Esmans EL (2006) Stainless steel electrospray probe: a dead end for phosphorylated organic compounds? J Chromatogr A 1104:209–221. <https://doi.org/10.1016/j.chroma.2005.12.004>
